# Aerobic bacteria associated with chronic suppurative otitis media in Angola

**DOI:** 10.1186/s40249-018-0422-7

**Published:** 2018-05-03

**Authors:** Fabian Uddén, Matuba Filipe, Åke Reimer, Maria Paul, Erika Matuschek, John Thegerström, Sven Hammerschmidt, Tuula Pelkonen, Kristian Riesbeck

**Affiliations:** 1 Clinical Microbiology, Department of Translational Medicine, Faculty of Medicine, Lund University, Malmö, Sweden; 2 ORL-department Hospital Josina Machel, Faculty of Medicine, Agostinho Neto University, Luanda, Angola; 3 Näshälsan Höllviken AB, Höllviken, Sweden; 4 EUCAST Development Laboratory, c/o Clinical Microbiology, Central Hospital, Växjö, Sweden; 5 Department of Molecular Genetics and Infection Biology, University of Greifswald, Greifswald, Germany; 6 Children’s Hospital, Helsinki University Hospital, Helsinki, Finland and University of Helsinki, Helsinki, Finland

**Keywords:** Chronic suppurative otitis media, Enterobacteria, Infection, Otitis media, *Proteus*, *Pseudomonas aeruginosa*

## Abstract

**Background:**

Chronic suppurative otitis media (CSOM) is an important cause of hearing loss in children and constitutes a serious health problem globally with a strong association to resource-limited living conditions. Topical antibiotics combined with aural toilet is the first-hand treatment for CSOM but antimicrobial resistance and limited availability to antibiotics are obstacles in some areas. The goal of this study was to define aerobic pathogens associated with CSOM in Angola with the overall aim to provide a background for local treatment recommendations.

**Methods:**

Samples from ear discharge and the nasopharynx were collected and cultured from 152 patients with ear discharge and perforation of the tympanic membrane. Identification of bacterial species was performed with matrix-assisted laser desorption/ionization-time of flight mass spectrometry and pneumococci were serotyped using multiplex polymerase chain reactions. Antimicrobial susceptibility testing was done according to EUCAST.

**Results:**

One hundred eighty-four samples from ear discharge and 151 nasopharyngeal swabs were collected and yielded 534 and 289 individual isolates, respectively. In all patients, correspondence rate of isolates from 2 ears in patients with bilateral disease was 27.3% and 9.3% comparing isolates from the nasopharynx and ear discharge, respectively. *Proteus* spp. (14.7%), *Pseudomonas aeruginosa* (13.2%) and *Enterococcus* spp. (8.8%) were dominating pathogens isolated from ear discharge. A large part of the remaining species belonged to *Enterobacteriaceae* (23.5%). Pneumococci and *Staphylococcus aureus* were detected in approximately 10% of nasopharyngeal samples. Resistance rates to quinolones exceeded 10% among *Enterobacteriaceae* and was 30.8% in *S. aureus*, whereas 6.3% of *P. aeruginosa* were resistant.

**Conclusions:**

The infection of the middle ear in CSOM is highly polymicrobial, and isolates found in nasopharynx do not correspond well with those found in ear discharge. Pathogens associated with CSOM in Angola are dominated by gram-negatives including *Enterobacteriaceae* and *P. aeruginosa,* while gram-positive enterococci also are common. Based on the results of antimicrobial susceptibility testing topical quinolones would be the preferred antibiotic therapy of CSOM in Angola. Topical antiseptics such as aluminium acetate, acetic acid or boric acid, however, may be more feasible options due to a possibly emerging antimicrobial resistance.

**Electronic supplementary material:**

The online version of this article (10.1186/s40249-018-0422-7) contains supplementary material, which is available to authorized users.

## Multilingual abstracts

Please see Additional file 1 for translation of the abstract into the six official working languages of the United Nations.

## Background

Chronic suppurative otitis media (CSOM) is a prolonged and often recurring bacterial infection of the middle ear defined by perforation of the tympanic membrane and otorrhoea lasting more than 2 weeks according to the World Health Organization (WHO), although a commonly used clinical definition is 6 weeks. The infection usually develops in early childhood, with a peak around 2 years of age, but can persist until adulthood [1, 2]. WHO estimates that 65 to 330 million people suffer from CSOM worldwide. The greatest burden of disease is found in low-income countries in Sub-Saharan Africa and Oceania, where incidence rates over 0.7% have been reported, with higher numbers among children under 5 years of age [1, 3]. Furthermore, certain ethnical groups are particularly affected, including the Inuit of Greenland, Native Americans and Aboriginal Australians [2]. Risk factors associated with CSOM include frequent episodes of acute otitis media (AOM), other respiratory tract infections, and traumatic tympanic rupture as well as factors correlating with resource-limited living conditions such as overcrowding, poor nutrition and hygiene, and chronic infectious diseases. Even if the pathogenesis is multifactorial, the clinical onset is frequently an episode of AOM complicated by tympanic membrane perforation and a subsequent superinfection of the middle ear with bacteria entering through the outer ear channel. Additionally, perforated tympanic membrane causes Eustachian tube dysfunction, allowing for pathogens to ascend to the middle ear through reflux of nasopharyngeal secretions [2, 4]. Formation of biofilm has been implicated to sustain the infection and reduce the efficacy of antibiotic treatment [5]. CSOM is an important cause of conductive as well as sensorineural hearing loss in children, which may be the result in more than 50% of patients. Moreover, facial nerve paralysis, sinus thrombosis, labyrinthitis, meningitis and brain abscesses are other rare complications. The WHO estimates that up to 28 000 yearly deaths can be attributed to CSOM on a global basis [1].

Microbiological findings in CSOM vary between studies. However, the bacterial spectrum most often identified in the CSOM-affected middle ear is dominated by *Pseudomonas aeruginosa, Staphylococcus aureus* and *Enterobacteriaceae* such as *Proteus* spp. and *Klebsiella pneumoniae* [1, 2, 4]. Anaerobic bacteria are commonly detected in studies of CSOM applying suitable anaerobic methods for isolation. Infections are often polymicrobial and a synergistic relationship between aerobes and anaerobes has been suggested [2, 6]. *Mycobacterium tuberculosis* is a rare cause of chronic infection of the middle ear, but tuberculous otomastoiditis has to be considered in a patient presenting with chronic ear discharge [7].

Being an important cause of pediatric hearing loss in developing countries that is potentially preventable, studies facilitating efficient care of CSOM are highly desirable. As the pathogenesis and varying microbiology of CSOM are not fully understood, it is of importance to investigate the prevalence of various pathogens in different areas [2]. Knowledge on the local incidence as well as spectrum of bacteria present and their antimicrobial susceptibility patterns is imperative for effective empirical treatment as well as contributing to the general understanding of the disease [6].

Luanda, the capital of Angola, has a population of approximately 6 million in 2014, and is one of the fastest growing cities in Africa, where a large part of the population lives in conditions lacking sanitation, fresh drinking water and with a high mortality rate among children under 5 years of age [8, 9]. Studies during 1981–1982 showed a CSOM prevalence of 2–4% in school children in Luanda [10]. Moreover, studies from 2011 reported CSOM in 4% of healthy children in Luanda and higher numbers in those with comorbidities such as HIV and tuberculosis [11, 12]. Studies on antimicrobial susceptibility of pathogens involved in CSOM from Angola are scarce, and generally do not regard the antibiotics commonly used to treat this particular infection. A high incidence of methicillin resistant *S. aureus* (MRSA) and carbapenemase-producing *Enterobacteriaceae* has, however, been reported [13, 14]. In the current study, we present microbiological findings from middle ear discharge and the nasopharynx in patients with CSOM in Luanda and three other provinces in Angola with the aim to provide a background for recommendations on the treatment of the disease in the public health care system.

## Methods

### Study design

The present study is a part of a project at the Ear, nose and throat (ENT)-department at Hospital Josina Machel (HJM) in Luanda to improve otitis media care and prevent hearing loss. AOM and CSOM are among the most frequent reasons for attending this clinic, which until 2016 was the only public ENT-clinic in Luanda. Clinical samples and patient information were collected from patients of all ages with CSOM at the outpatient section of the HJM ENT-clinic and at health care centers in three other Angolan provinces (Lunda Sul, Namibe, Zaire) from January to December 2016. CSOM was defined as perforation of the tympanic membrane confirmed by otoscopy and purulent ear discharge lasting more than 14 days.

### Sampling and culture conditions

Sampling for microbiological cultures was conducted by experienced clinicians using standard techniques. After cleaning the auditory channel with 70% ethanol, ear discharge samples were collected with a swab. For nasopharyngeal sampling, a swab was introduced into nasopharynx through the nostril crossing the choana until it touched the wall of the nasopharynx. Samples from both loci were collected in skim milk-tryptone-glucose-glycerol (STGG) medium and stored at − 70 °C at the Public Health Laboratory (Luanda) prior to transport to the Riesbeck laboratory (Malmö, Sweden). Clinical specimens were cultured on hematin agar, Columbia CNA agar (Oxoid, Hampshire, UK), and UriSelect agar supplemented with vancomycin (Bio-Rad, Hercules, CA) and incubated at 35.5 °C in 5% CO_2_ (hematin and Columbia CNA agar) or at aerobic conditions (UriSelect) for 16–18 h.

### Species identification and serotyping of pneumococci

Bacterial species identification was done by Matrix-assisted laser desorption/ionization – time of flight mass spectrometry (MALDI-TOF MS) [15]. Briefly, bacteria from a single colony of each isolate were applied to a MALDI target plate (Bruker Daltonics, Bremen, Germany) in duplicates and were overlaid with 1 μl HCCA matrix (Bruker Daltonics) and let dry completely. Mass spectra were then acquired with a microflex MALDI-TOF mass spectrometer with flexControl software (Bruker Daltonics, Bremen, Germany) using default settings (mass range of spectra, m/z 2000 to 20 000 in linear positive-ionization mode), and species were identified using the MALDI Biotyper 4.1 software with Bruker taxonomy library (*N* = 6903) (Bruker Daltonic). *Streptococcus pneumoniae* were serotyped using multiplex polymerase chain reaction (mPCR) [16]. Briefly, DNA was prepared from each pneumococcal isolate by boiling bacteria in Tris-EDTA-buffer and thereafter analysed in 6 sequential mPCRs containing, in total, 32 primer pairs identifying genes specific to different serotypes or groups of serotypes. A primer pair identifying the pneumococcus *cpsA* gene was included in all reactions as internal positive control indicating successful reaction. Primer sequences and the distribution of primer pairs to the different reactions, as well as PCR cycling conditions are presented in Additional file 2. If a pneumococcal serotype could not be identified by mPCR or further serotyping was needed to distinguish individual serotypes the Pneumotest Latex Kit and Neufeldt Antisera (Statens Seruminstitut, Copenhagen, Denmark), both based on the Quellung-reaction of capsular swelling, were used according to the manufacturer’s instructions.

### Antimicrobial susceptibility testing

Antimicrobial susceptibility testing was performed with disc diffusion according to European Committee on Antimicrobial Susceptibility Testing for all antibiotics except colistin, for which broth microdilution was performed on MICRONAUT-S plates (Merlin Diagnostika, Bornheim, Germany) according to the manufacturer’s instructions. Susceptibility testing was done on the species that are generally regarded as being relevant for the pathogenesis of CSOM or AOM and the results were interpreted according to Breakpoint Tables of the European Committee on Antimicrobial Susceptibility Testing [17]. A number of isolates of the relevant species were not available for susceptibility testing. These isolates were, however, missing at random.

### Statistical analysis

Descriptive statistics are used to present demographics, species distribution and microbial susceptibility. All data were computerized and analysed using Microsoft Excel version 15.37 (Microsoft, Redmond, WA) and are presented as absolute numbers and percentages.

## Results

### CSOM is mainly found in children less than 12 years of age

**Fig. 1:**
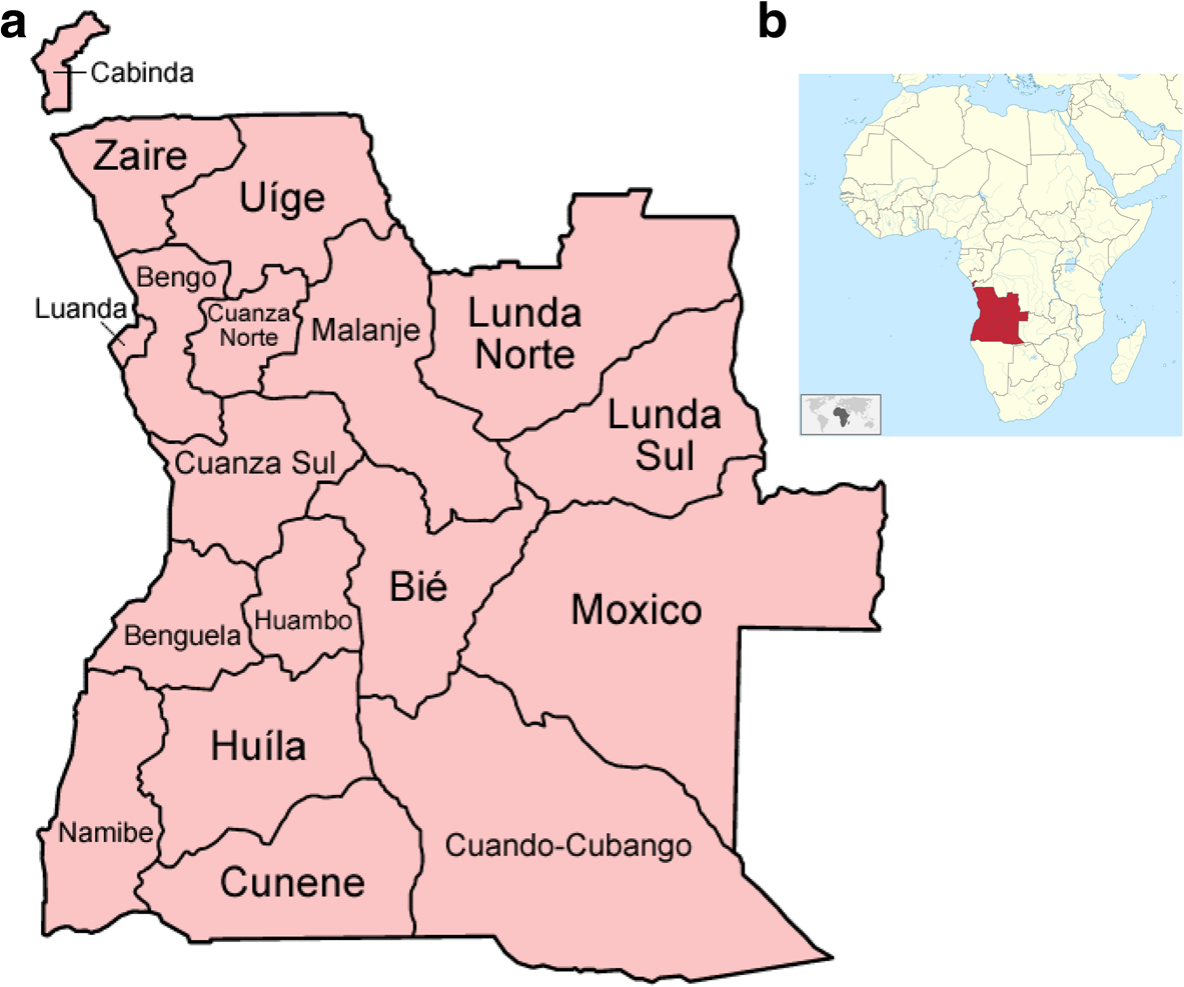
**a** Map of Angola and its provinces [39] (by Golbez used under CC BY 2.5, https://creativecommons.org/licenses/by/2.5/). **b** Location map of Angola in Africa [40] (by TUBS used under CC BY-SA 3.0, https://creativecommons.org/licenses/by-sa/3.0/)

In total, 152 patients with tympanic membrane perforation and ear discharge lasting more than 14 days, attending the ENT-clinic or health care centers, were included in the study. A majority were from Luanda (77%; *N =* 111), whereas 17% (*N* = 24), 5% (*N* = 7) and 1% (*N* = 2) of patients were from Lunda Sul, Zaire and Namibe, respectively (Fig. 1). For 8 individuals, information about enrolment site was missing. Patient age ranged from 0 to 77 years and the median age was 12.6 years. Sixty-two percent (*N* = 91) of the patients were male while 38% (*N* = 56) were female. A list of all patients, including demographical data and a listing of all microbiological findings is supplied in Additional file 3.

### Co-colonization by several different species is common in the CSOM-affected middle ear

Samples from ear discharge from one or both ears (*N* = 184) and nasopharyngeal swabs (*N* = 151) were collected. A total of 823 microbes were identified, 534 in samples from ear discharge and 289 in nasopharyngeal samples. The mean number of isolates per sample was slightly higher in ear discharge samples than in nasopharyngeal samples (2.9 vs. 2.4, respectively). Only 16 ear samples yielded a single isolate as compared to 55 nasopharyngeal swabs. Of 187 isolates recovered from the middle ears in patients with bilateral disease, 52 (27.3%) were isolates of the same species present in both ears of the same individual. On the other hand, 9.3% (*N* = 27) of the nasopharyngeal isolates corresponded to an isolate simultaneously present in ear discharge from the same patient. The number of samples and isolates with regard to age group are described in Table 1.

**Table 1:** Demographics of the study population and numbers of samples collected and pathogens isolated with regard to age group

Age group (years)	*N* (%)	Gender (male/female)^1^	Ear discharge samples		Nasopharyngeal samples		Total		Corresponding isolates^3^	
			*N*	Isolates^2^	*N*	Isolates^2^	*N*	Isolates^2^	Ear/Ear	Ear/Nph
< 5	34 (22)	20/14	44	124 (2.8 [0–5])	34	104 (3.1 [1–6])	78	194 (2.5 [0–6])	10 (18.2)	7 (10)
5–9	23 (15)	15/8	26	81 (3.1 [0–6])	23	52 (2.3 [0–3])	49	117 (2.4 [0–6])	7 (36.8)	3 (8.3)
10–14	24 (16)	16/8	31	97 (3.1 [1–7])	24	51 (2.1 [0–5])	55	144 (2.6 [0–7])	15 (33.3)	6 (12.8)
≥ 15	55 (36)	33/22	66	184 (2.8 [1–6])	54	109 (2.0 [0–4])	120	286 (2.4 [0–6])	18 (29.5)	11 (10.8)
No age data	16 (11)	7/4	17	48 (2.8 [1–4])	16	45 (2.8 [1–4])	33	96 (2.9 [1–4])	2 (28.6)	0
Total	152	91/56	184^4^	534 (2.9 [0–7])	151^5^	361 (2.4 [0–6])	335	823 (2.5 [0–7])	52 (27.3)	27 (9.3)

No data about age or gender was available for 16 and 5 patients, respectively

^1^ Gender data was missing for 5 patients

^2^ Presented as *N* (mean number of isolates per sample [range])

^3^ Number of species present in two ear discharge samples, or in samples from ear discharge and the nasopharynx, from the same patient. Presented as N (%)

^4^ Bilateral samples were collected from 32 patients

^5^ No nasopharyngeal sample was collected from 1 patient

### *Proteus* spp.*, Pseudomonas aeruginosa* and enterococci dominate in CSOM

A summary of the microbiological findings is presented in Table 2. In ear discharge samples, 87 different species were identified; *Proteus* spp. (14.7%), *P. aeruginosa* (13.2%) and *Enterococcus* spp. (8.8%) were, however, dominating. Other *Enterobacteriaceae* constituted a large group, representing 23.5% of isolates, in which *Providencia* spp., *Morganella morganii, Citrobacter* spp. and *Klebsiella* spp. were the most prevalent bacteria. In contrast, *Pseudomonas* spp. other than *P. aeruginosa* (16.0%), pneumococci (10.9%) and *S. aureus* (8.2%) were dominating in nasopharyngeal samples, beside the abundant occurence of coagulase-negative staphylococci (CoNS) (30.7%). Of 34 pneumococcal isolates, 35.3% (*N* = 12) were identified as serotypes included in the 13-valent pneumococcal conjugate vaccine (PCV13). Serotype 19F (*N* = 4) was the most common serotype followed by serotype 6A (*N* = 3) and non-PCV13 serotype 17F (*N* = 3) (Table 3).

**Table 2:** Microbiological findings in samples from ear discharge and the nasopharynx

Isolates from ear discharge (*N* = 534)		Isolates from nasopharynx (*N* = 289)	
Pathogen	*N* (%)	Pathogen	*N* (%)
*Proteus* spp.	79 (14.7)	CoNS	90 (30.7)
*Pseudomonas aeruginosa*	71 (13.2)	*Pseudomonas* spp.	47 (16.0)
*Enterococcus* spp.	47 (8.8)	*Streptococcus pneumoniae*	32 (10.9)
*Providencia* spp.	42 (7.8)	*Staphylococcus aureus*	24 (8.2)
CoNS	39 (7.3)	*Proteus* spp.	12 (4.1)
*Corynebacterium* spp.	31 (5.8)	*Moraxella catarrhalis*	10 (3.4)
*Morganella morganii*	24 (4.5)	*Enterococcus* spp.	10 (3.4)
*Alcaligenes faecalis*	19 (3.5)	*Arthrobacter* spp.	8 (2.7)
*Citrobacter* spp.	18 (3.4)	*Acinetobacter* spp.	6 (2.0)
*Klebsiella* spp.	16 (3.0)	*Alcaligenes faecalis*	6 (2.0)
*Arthrobacter* spp.	15 (2.8)	*Haemophilus influenzae*	5 (1.7)
*Staphylococcus aureus*	14 (2.6)	*Corynebacterium* spp.	4 (1.4)
Other *Streptococcus* spp.	12 (2.2)	Fungi	4 (1.4)
Fungi	12 (2.2)	*Pantoea agglomerans*	4 (1.4)
*Kerstersia gyiorum*	12 (2.2)	*Streptococcus pyogenes*	3 (1.0)
*Escherichia coli*	11 (2.0)	*Enterobacter* spp.	3 (1.0)
*Enterobacter* spp.	11 (2.0)	Other^2^	21 (7.2)
*Pseudomonas* spp.	10 (1.9)	No growth	4 (1.4)
*Achromobacter* spp.	7 (1.3)		
*Streptococcus pyogenes*	6 (1.1)		
Other^1^	38 (7.1)		
No growth	3 (0.6)		

Pathogens representing less than 1% of isolates in each group have been pooled as “Other”. CoNS = coagulase-negative staphylococci

^1^ Included pathogens listed from most to least prevalent: *Stenotrophomonas maltophilia*, *Acinetobacter* spp., *Gemella morbillorum*, *Bordetella trematum*, *Globicatella sulfidifaciens*, *Aeromonas caviae*, *Escherichia hermannii*, *Streptococcus pneumoniae*, *Aerococcus viridans*, *Arcanobacterium haemolyticum*, *Dermabacter hominis*, *Kocuria* spp., *Microcuccus* spp., *Weeksella virosa*, *Raoultella ornithinolytica*, *Serratia marcescens*, *Lactococcus lactis*, *Weisella confusa*, *Neisseria meningitidis*

^2^ Included pathogens listed from most to least prevalent: *Klebsiella* spp., *Aerococcus viridans*, *Lactococcus lactis*, *Leuconostoc* spp., *Macrococcus caseolyticus*, *Moraxella nonliquefaciens*, *Raoultella ornithinolytica*, *Streptococcus* spp., *Citerobacter koseri*, *Morganella morganii*, *Serratia marcescens*, *Dietzia maris*, *Lactobacillus salivarius*, *Shewanella putrefaciens*, *Stenotrophomonas maltophilia*

**Table 3:** Pneumococccal serotypes identified in 34 isolates

Serotype	*N*	(%)
19F*	4	(11.8)
6A*	3	(8.8)
17F	3	(8.8)
16	2	(5.9)
21	2	(5.9)
38	2	(5.9)
11A	2	(5.9)
23B	2	(5.9)
NT	2	(5.9)
4*	1	(2.9)
18C*	1	(2.9)
19A*	1	(2.9)
23F*	1	(2.9)
6B*	1	(2.9)
15A	1	(2.9)
15C	1	(2.9)
19B	1	(2.9)
12	1	(2.9)
13	1	(2.9)
20	1	(2.9)
34	1	(2.9)
Total	34	(100)
Total PCV13-serotypes	12	(35.3)

Asterisks indicate serotypes included in the 13-valent pneumococcal conjugate vaccine (PCV13). NT = non-typeable

### Most pathogenic bacteria in CSOM are susceptible to fluoroquinolones

Susceptibility patterns for gram-negative and gram-positive bacteria are presented in Table 4 and Table 5, respectively. The majority of tested isolates were susceptible to quinolones (i.e.*,* ciprofloxacin and norfloxacin), and resistance rates in bacteria relevant to CSOM ranged from 6.3 to 30.8% for *P. aeruginosa* and *S. aureus,* respectively*.* Several isolates within *Enterobacteriaceae* were resistant against aminoglycosides (range 6.9–25.8%), slightly more increased resistance against gentamicin as compared to tobramycin. In contrast, resistance against aminoglycosides was lower for *P. aeruginosa* and higher in *S. aureus*. Almost half of isolated *Enterobacteriaceae* (42.6%), and all *P. aeruginosa*, were resistant against chloramphenicol. A high resistance against trimethoprim-sulfamethoxazol was observed in *Enterobacteriaceae* while more than 90% were susceptible to cefotaxime. We also found that a high proportion of *S. aureus* (53.8%) were methicillin-resistant (MRSA). Furthermore, most pneumococci were resistant against benzylpenicillin (53.1%) and trimethoprim-sulfamethoxazol (78.1%).

**Table 4:** Susceptibility of gram-negative bacterial species to selected antimicrobial agents

Antimicrobial agent	Susceptibility^1^	*Proteus* spp. (*N* = 89)^2^		non-*Proteus Enterobacteriaceae* (*N* = 131)^3^		*P. aeruginosa* (*N* = 63)^4^		*H. influenzae* (*N* = 5)		*M. catarrhalis* (*N* = 9)	
		*N*	(%)	*N*	(%)	*N*	(%)	*N*	(%)	*N*	(%)
Amoxicillin	S							4	(80)		
	R							1	(20)		
Piperacillin-tazobactam	S	88	(98.9)	125	(95.4)	61	(96.8)				
	I			5	(3.8)						
	R	1	(1.1)	1	(0.8)	2	(3.2)				
Cefotaxime	S	88	(98.9)	121	(92.3)			5	(100)	9	(100)
	I			1	(0.8)						
	R	1	(1.1)	9	(6.9)						
Ceftazidime	S	88	(1.1)	122	(93.1)	62	(98.4)				
	I			2	(1.5)						
	R	1	(1.1)	7	(5.3)	1	(1.6)				
Imipenem	S	89	(100)	122	(93.1)	60	(95.2)				
	I			9	(6.9)	3	(4.8)				
Meropenem	S	89	(100)	131	(100)	52	(82.5)	5	(100)	9	(100)
	I					11	(17.5)				
Ciprofloxacin	S	76	(85.4)	105	(80.2)	58	(92.1)	5	(100)	9	(100)
	I	3	(3.4)	7	(5.3)	1	(1.6)				
	R	10	(11.2)	19	(14.5)	4	(6.3)				
Gentamicin	S	63	(70.8)	106	(80.9)	58	(92.1)				
	I	3	(3.4)	3	(2.3)	1	(1.6)				
	R	23	(25.8)	22	(16.8)	4	(6.3)				
Tobramycin	S	72	(80.9)	109	(83.2)	59	(93.7)				
	I	5	(5.6)	13	(9.9)						
	R	12	(13.5)	9	(6.9)	4	(6.3)				
Tetracycline	S							4	(80)	7	(77.8)
	R							1	(20)	2	(22.2)
Trimethoprim-sulfamethoxazole	S	31	(34.8)	71	(54.2)			2	(40)	1	(11.1)
	I	2	(2.2)	2	(1.5)					1	(11.1)
	R	56	(62.9)	58	(44.3)			3	(60)	7	(77.8)
Chloramphenicol	S			27	(57.4)						
	R			20	(42.6)	42	(100)				
Colistin	S			26^3^	(55.3)	40^4^	(95.2)				
	R	70^2^	(100)	21^3,5^	(44.7)	2^4,6^	(4.8)				

Isolates grouped as non-*Proteus Enterobacteriaceae* were 38 *Providencia* spp., 25 *Morganella morganii*, 18 *Klebsiella* spp., 18 *Citrobacter* spp., 13 *Enterobacter* spp., 13 *Escherichia* spp., 3 *Pantoea agglomerans,* 2 *Serratia marcescens* and 1 *Raoultella ornithinolytica*

^1^ S, susceptible; I, intermediate; R, resistant

^2^ Susceptibility testing for *Proteus* spp. against colistin was done with 70 randomly selected isolates

^3^ Susceptibility testing for non-*Proteus Enterobacteriaceae* against chloramphenicol and colistin was done with 47 randomly selected isolates

^4^ Susceptibility testing for *P. aeruginosa* against chloramphenicol and colistin was done with 42 randomly selected isolates

^5^ All MIC ≥64 mg/L including *Providencia*, *Morganella*, *Enterobacter* and *Serratia* spp.

^6^ All MIC = 4 mg/L

**Table 5:** Susceptibility of gram-positive bacterial species to selected antimicrobial agents

Antimicrobial agent	Susceptibility^1^	*S. pneumoniae*		*S. pyogenes*		*S. aureus*	
		(*N* = 32)		(*N =* 9)		(*N* = 13)	
		*N*	(%)	*N*	(%)	*N*	(%)
Benzylpenicillin	S	15	(46.9)	9	(100)		
	R	17	(53.1)				
Cefoxitin^2^	S					6	(46.2)
	R					7	(53.8)
Norfloxacin^3^	S	32	(100)			9	(69.2)
	R					4	(30.8)
Tobramycin	S					4	(30.8)
	R					9	(69.2)
Erythromycin	S	32	(100)	9	(100)	12	(92.3)
	R					1	(7.7)
Clindamycin	S	32	(100)	9	(100)	11	(84.6)
	I					1	(7.7)
	R					1	(7.7)
Tetracycline	S	24	(75)	3	(33.3)		
	I	1	(3.1)				
	R	7	(21.9)	6	(66.7)		
Fusidic acid	S					13	(100)
Rifampicin	S	27	(84.4)				
	R	5	(15.6)				
Trimethoprim-sulfamethoxazole	S	6	(18.8)			13	(100)
	I	1	(3.1)				
	R	25	(78.1)				

^1^ S, susceptible; I, intermediate; R, resistant

^2^ Screening substance for methicillin resistance

^3^ Screening substance for quinolone resistance

## Discussion

It is a well known fact that infection of the middle ear in CSOM is usually polymicrobial, and the present study further confirms this as single isolates were identified in very few ear discharge samples [2]. In fact, a wide range of different species was identified. The high number of bacterial strains and degree of co-colonization reported, in both ear discharge and nasopharyngeal samples, can possibly be attributed to a higher specificity of MALDI-TOF MS as compared to conventional microbiological identification methods [15]. The slight difference in number of males and females in the current study is unclear, but may be due to differences in care-seeking behaviour, as CSOM has been observed to affect gender equally [2]. However, a predominance of males was shown in an earlier report from the same clinic [18].

The nasopharyngeal microbiome has been proposed as a reservoir for pathogens involved in CSOM, and culture from this locus may grant important clinical information during the disease [19]. However, we found a low degree of correspondence between organisms present in the nasopharynx and middle ear discharge in the same patients. On the other hand, > 25% of the isolates from ear samples in patients with bilateral disease corresponded between the ears. It is possible that this is due to spread via the nasopharynx as the Eustachian tube is dysfunctional in CSOM, but may also be due to invasion from the outer ear canal by the same species in both ears [4]. The role of the nasopharyngeal flora in the pathogenesis and sustaining of CSOM is a field that needs to be further explored.

In accordance with our results, Taipale et al. [12] previously found *Proteus* and *P. aeruginosa* to be the most prevalent bacteria in 18 patients with CSOM in Luanda. In a Kenyan study, enterococci (28%) were reported as a common pathogen in CSOM together with *Proteus*, *S. aureus* and *P. aeruginosa*, representing 32%, 12% and 11% of isolates, respectively [20]. Orji et al. [21] and Afolabi et al. [22] both found that *P. aeruginosa* is the dominating species in Nigeria followed by *S. aureus* and *Klebsiella,* respectively. On the other hand, Chirwa et al. [23] defined *Proteus* spp. as dominating followed by *P. aeruginosa* in Malawi. Thus, our results on the most prevalent pathogens in CSOM are in concordance with other recent studies from sub-Saharan Africa, although there seem to be geographical differences in the proportions between the species present.

An interesting finding was 19 isolates of *Alcaligenes faecalis,* which may be due to the occasional custom of filling the external meatus of the ear with bird droppings to prevent discharge, that we previously reported [24]. Furthermore, 12 isolates of *Kerstersia gyiorum* was detected. This gram-negative species belongs to the *Alcaligenaceae* family and has previously been found in CSOM with treatment failure due to antimicrobial resistance [25]. We did not have the possibility to study anaerobes in the present work due to methods used for sampling, transportation and microbiological diagnostics, although there is support for the presence of these bacteria in a majority of CSOM infections [6]. The high prevalence of CoNS and *Corynebacterium* spp., that both belong to the skin microbiome of the external auditory channel, was most likely contamination during collection [26]. It is possible that other isolated species also represent contamination from adjacent anatomical sites, and thus more studies are needed to elucidate the rich bacterial spectrum found in CSOM applying methods that enable specific species identification.

Aural toilet combined with empirical antibiotic therapy with topical quinolone antibiotics is the recommended first-hand treatment for uncomplicated CSOM although topical aminoglycosides, polymyxins or chloramphenicol are also used. Moreover, antiseptic topical agents such as aluminium acetate, acetic acid or boric acid may be effective and more feasible in resource-limited conditions due to their lower cost and availability [27–29]. The susceptibility patterns of the most frequently isolated species in this study suggest that quinolones or aminoglycosides may be more successful in clearing infection than colistin or chloramphenicol, the latter being the currently most used topical antibiotic for CSOM in Angola. Quinolones have previously been shown to be more effective than aminoglycosides, and would also be the preferred choice of the two drugs due to the potential ototoxicity of aminoglycosides [2]. However, resistance rates over 10% were observed for quinolones, which highlights the need to continuously determine antimicrobial susceptibility patterns. Other findings of interest regarding the general occurrence of antimicrobial resistance are high rates of MRSA, aminoglycoside-resistance among gram-negatives and penicillin non-susceptible pneumococci. On the other hand, only a small number of *Enterobacteriaceae* were resistant to cefotaxime, i.e. probable carriage of extended-spectrum beta-lactamases, and carbapenems which has previously been reported as common in Luanda [14].

Considering the risk for antimicrobial resistance to all topical antibiotics tested, and the fact that these antibiotics may not be readily available in resource-limited settings, the use of topical antiseptics should be explored. Treatment with one-off application of boric acid powder has been reported to be as effective as topical ciprofloxacin while treatment with aluminium acetate has showed similar results compared to topical aminoglycosides [2, 29]. In a randomized controlled trial in Tanzania the treatment choice for CSOM in children was daily aural toilet and topical boric acid in alcohol solution [30]. Furthermore, Youn et al. [31] showed a high bactericidal effect of aluminium acetate and acetic acid against MRSA and quinolone-resistant *P. aeruginosa*.

Within the framework of the current project at HJM, a film has been produced informing about the condition to improve care of CSOM. Here ear care by “dry mopping” and protecting the ear from contamination is described in detail. It is available online and is planned to be presented in social media and television in Angola [32]. Such information and improved treatment in health care centers and hospitals might reduce the burden of CSOM and its complications.

As CSOM is generally preceded by an episode of AOM, which is predominantly caused by pneumococci, non-typeable *Haemophilus influenzae* or *Moraxella catarrhalis* [33]*,* an important aspect of CSOM prevention is the reduction of AOM. In fact, incidence rates of AOM in western sub-Saharan Africa has been estimated to be over 40%, with a majority of cases occurring in children under 5 years of age [3]. Some studies suggest that the use of PCV may have a positive effect on the all-cause AOM incidence, which may lead to a subsequently reduced CSOM incidence [34, 35]. PCV13 was introduced in Angola in 2013 and WHO estimates that the proportion of newborns who received three vaccine doses has risen from 9% in 2013 to 58% in 2015 and 2016 [36]. Although a small number of pneumococci were isolated, our results show that vaccine serotypes are present in Angola indicating that any obvious serotype replacement, which is a well-documented effect of PCV in many areas of the world, has not occurred, at least not in the mixed age group studied [37, 38]. Considering these numbers and the possible effects on otitis media, a higher degree of vaccine coverage is desirable in Angola. However, further studies are required on this topic.

## Conclusions

The results of the current study largely agree with other studies concerning the dominating pathogens found in CSOM although a greater number of individual species were identified. *Proteus* spp., *P. aeruginosa* and enterococci were the most frequently identified bacteria in ear discharge. Based on the susceptibility testing performed the best choices for topical antibiotic treatment of CSOM in the current outpatients setting would be quinolones. However, considering the risk for unsuccessful treatment due to antimicrobial resistance, topical antiseptic agents should be considered as the first-hand choice for treatment of CSOM in Angola.

## Additional files

Additional file 1: Multilingual abstracts in the six official working languages of the United Nations. (PDF 675 kb)

Additional file 2: Primer list and PCR cycling conditions. (PDF 126 kb)

Additional file 3: Full patient list. (PDF 258 kb)

## Abbreviations

AOM: Acute otitis media
CoNS: Coagulase-negative staphylococci
CSOM: Chronic suppurative otitis media
ENT: Ear, nose and throat
HJM: Hospital Josina Machel
MALDI-TOF MS: Matrix-assisted laser desorption/ionization – time of flight mass spectrometry
mPCR: Multiplex polymerase chain reaction
MRSA: Methicillin-resistant *S. aureus*
PCV: Pneumococcal conjugate vaccine
WHO: World Health Organization
