# Supplementary material for: Aerobic bacteria associated with chronic suppurative otitis media in Angola
Source: Infect Dis Poverty. 2018 May 3;7:42. doi: 10.1186/s40249-018-0422-7 (PMC5932871; doi:10.1186/s40249-018-0422-7)
Supplement: Supplementary file 1 — Multilingual abstracts in the six official working languages of the United Nations. (PDF 675 kb) [file 40249_2018_422_MOESM1_ESM.pdf]

Translation of the abstract into the six official working languages of the United Nations

البكتيريا المسببة لالتهاب الأذن الوسطى الصديدي المزمن - دراسة لمعدل انتشار المرض في نقطة زمنية محددة في أنغولا.

فابيان أودين، ماتوبا فيليبي، آكي ريمر، ماريا بول، إريكا ماتوشيك، جون ثيغريستروم، سفين هامرشميدت، تولا بيلكونين، كريستيان ريسبيك

الملخص

الخلفية: التهاب الأذن الوسطى الصديدي المزمن (CSOM) هو أحد أهم أسباب فقدان السمع لدى الأطفال؛ وهو يمثل مشكلة صحية عالمية خطيرة ترتبط ارتباطًا وثيقًا بالظروف المعيشية محدودة الموارد. خط العلاج الأول لالتهاب الأذن الوسطى الصديدي المزمن هو المضادات الحيوية الموضعية إلى جانب غسل الأذن؛ إلا أن مقاومة البكتيريا لمضادات الميكروبات، وقلة توافر المضادات الحيوية تقف عقبة أمام العلاج في بعض المناطق. هدف هذه الدراسة هو تحديد الأجسام الهوائية الممرضة المرتبطة بالتهاب الأذن الوسطى الصديدي المزمن في أنغولا؛ والهدف الأعم هو وضع خلفية أساسية من أجل الخروج بتوصيات محلية للعلاج.

الوسائل: جُمعت واستنبتت عينات من إفرازات الأذن ومن البلعوم الأنفي من 152 مريضًا يعاني من إفرازات الأذن وانثقاب غشاء طبلة الأذن. تم التعرف على الفصائل البكتيرية باستخدام مطياف الكتلة بالتفاظ/تأين الليزر المعزز بمصفوفات لقياس زمن الانتقال الأيوني؛ وتم تحديد النمط المصلي لبكتيريا المكورات الرئوية باستخدام تفاعلات البوليمراز التسلسلية المتعددة. أجري اختبار الحساسية لمضادات الميكروبات وفقًا لمعايير EUCAST.

النتائج: جُمع مئة وأربع وثمانون عينة من إفرازات الأذن، و151 من مسحات البلعوم الأنفي؛ وقد أنتجت هذه العينات 534 و289 عينة بكتيرية معزولة، على التوالي. لدى جميع المرضى، كان معدل تطابق العينات المعزولة من أذنين في المرضى المصابين بالصورة ثنائية الجانب من المرض هو 27.3% و9.3% وذلك بمقارنة العينات المعزولة من البلعوم الأنفي ومن إفرازات الأذن، على التوالي. كانت فصيلة المتقلبة (14.7%)، وبكتيريا الزائفة الزنجارية (13.2%) وفصيلة المكورات المعوية (8.8%) هي الأجسام الممرضة الأساسية التي تم عزلها من إفرازات الأذن. كان جزء كبير من الفصائل المتبقية ينتمي لفصيلة الأمعائيات (23.5%). تم اكتشاف بكتيريا المكورات الرئوية وبكتيريا المكورات العنقودية الذهبية في حوالي 10% من عينات البلعوم الأنفي. فاقت معدلات مقاومة الكينولونات 10% لفصيلة الأمعائيات وبلغت 30.8 لبكتيريا المكورات العنقودية الذهبية، بينما كان 6.3% من بكتيريا الزائفة الزنجارية مقاومًا.

الاستنتاجات: تُعد عدوى الأذن الوسطى في مرض التهاب الأذن الوسطى الصديدي المزمن عدوى متعددة الميكروبات إلى حد كبير؛ ولا تتطابق العينات المعزولة المأخوذة من البلعوم الأنفي كثيرًا مع تلك الموجودة في إفرازات الأذن. الأجسام الممرضة المرتبطة بالتهاب الأذن الوسطى الصديدي المزمن في أنغولا هي في أغلبها من الأجسام سالبة الغرام، بما فيها الأمعائيات والزائفة الزنجارية، بينما تنتشرالمكورات المعوية موجبة الغرام هي الأخرى. استنادًا إلى نتائج اختبار الحساسية لمضادات الميكروبات؛ فمن الممكن أن تكون الكينولونات الموضعية هي المضادات الحيوية المفضلة لعلاج التهاب الأذن الوسطى الصديدي المزمن في أنغولا. ومع ذلك، فقد تكون المطهرات الموضعية مثل أسيتات الألومنيوم أو حمض الأسيتيك أو حمض البوريك خيارات أكثر فعالية بسبب احتمال ظهور مقاومة بكتيرية لمضادات الميكروبات.

Translated from English version into Arabic by Salma Anwar, proofread by Hesham Abdullah, through

TRANSLATORS
WITHOUT BORDERS

安哥拉地区与慢性化脓性中耳炎相关的有氧病原体

Fabian Uddén, Matuba Filipe, Åke Reimer, Maria Paul, Erika Matuschek, John Thegerström, Sven Hammerschmidt, Tuula Pelkonen, Kristian Riesbeck

摘要

引言：慢性化脓性中耳炎（CSOM）是儿童听力丧失的重要原因，是全球范围内重要的公共卫生问题，与资源匮乏的生活条件密切相关。局部使用抗生素联合耳内盥洗治疗是 CSOM 的首选治疗方案，但是抗生素耐药性以

及抗生素的限制性使用阻碍了该方案在某些地区的实施。本研究目的是确认与安哥拉地区 CSOM 相关的需氧病原体，为当地治疗方案提供参考。
方法：收集 152 例有耳部分泌物或鼓膜穿孔患者的耳分泌物和鼻咽拭子。使用基质辅助激光解吸/电离-飞行时间质谱鉴定细菌种类，使用多重 PCR 方法对肺炎链球菌进行血清型分型。根据 EUCAST 进行抗微生物药物敏感性测试。
结果：共收集 184 份耳分泌物和 151 份鼻咽拭子样本，分别获得 534 份和 289 份分离株。在所有双侧发病患者中，2 耳分离物符合率为 27.3%，鼻咽拭子和耳分泌物的符合率为 9.3%。变形杆菌属（14.7%）、铜绿假单胞菌（13.2%）和肠球菌属（8.8%）是耳分泌物分离出的主要病原体。其余大部分属于肠杆菌科（23.5%）。在大约 10%的鼻咽样本中检测到肺炎链球菌和金黄色葡萄球菌。喹诺酮类药物对肠杆菌科的耐药率超过 10%，对金黄色葡萄球菌和铜绿假单胞菌的耐药率分别为 30.8%和 6.3%。
结论：CSOM 的中耳感染是高度多菌性的，并且鼻咽样本的分离物与耳分泌物中的分离物不同。安哥拉地区与 CSOM 相关的病原体主要为革兰氏阴性菌，包括肠杆菌科和铜绿假单胞菌，革兰氏阳性肠球菌也很常见。抗菌药物敏感性试验的结果显示，喹诺酮类药物是安哥拉地区 CSOM 的首选抗生素，然而，由于可能出现的抗微生物药物耐药性，局部防腐剂如乙酸铝、乙酸或硼酸可能是更好的选择。

Translated from English version into Chinese by Xue-Jiao Teng, edited by Pin Yang

CHINESE CENTER FOR DISEASE CONTROL AND PREVENTION
NATIONAL INSTITUTE OF PARASITIC DISEASES

Bactéries aérobies associées à l'otite moyenne suppurée chronique en Angola

Fabian Uddén, Matuba Filipe, Åke Reimer, Maria Paul, Erika Matuschek, John Thegerström, Sven Hammerschmidt, Tuula Pelkonen, Kristian Riesbeck

Résumé
Contexte: L'otite moyenne suppurante chronique (OMSC) est une cause importante de perte d'audition chez les enfants et un problème grave de santé publique dans le monde, notamment dans les régions où les ressources sont limitées. Son traitement de première intention est une antibiothérapie topique associée à une toilette auriculaire, mais il se heurte à des résistances aux antimicrobiens et à la disponibilité limitée des antibiotiques dans certaines régions. Le but de notre étude était d'identifier les agents pathogènes aérobies associés à l'OMSC en Angola, afin de jeter les bases de recommandations de traitement locales.
Méthodes: Des échantillons de sécrétions auriculaires et nasopharyngées prélevés sur 152 patients présentant un écoulement auriculaire et une perforation du tympan ont été recueillis et mis en culture. Les espèces bactériennes ont été identifiées par spectrométrie de masse à temps de vol par désorption-ionisation laser assistée par matrice et le sérotype des pneumocoques a été déterminé par réaction en chaîne à la polymérase multiplexe. Des antibiogrammes ont été réalisés selon la méthode de l'EUCAST.
Résultats: Nous avons recueilli 184 échantillons de sécrétions auriculaires et 151 écouvillonnages nasopharyngés, qui ont donné respectivement 534 et 289 isolats individuels. Pour tous les patients, le taux de correspondance des isolats des deux oreilles des patients souffrant d'une atteinte bilatérale était de 27,3 %, et celui des comparaisons entre les sécrétions nasopharyngées et auriculaires de 9,3 %. *Proteus* spp. (14,7 %), *Pseudomonas aeruginosa* (13,2 %) et *Enterococcus* spp. (8,8 %) étaient les pathogènes dominants dans les prélèvements auriculaires. Les autres espèces étaient, pour une grande part, des entérobactéries (23,5 %). Des pneumocoques et *Staphylococcus aureus* ont été décelés dans environ 10 % des

échantillons nasopharyngés. Le taux de résistance aux quinolones dépassait 10 % parmi les entérobactéries et atteignait 30,8 % chez *Staphylococcus aureus*, tandis que 6,3 % des isolats de *P. aeruginosa* étaient résistants.

Conclusions: L'infection de l'oreille moyenne dans l'OMSC est polymicrobienne au plus haut point et les isolats identifiés dans le nasopharynx ne coïncident pas bien avec ceux des sécrétions auriculaires. Les agents pathogènes associés à l'OMSC en Angola sont principalement à Gram négatif, avec notamment des entérobactéries et *P. aeruginosa*, mais les entérocoques à Gram positif sont également courants. Les antibiogrammes suggèrent que les quinolones topiques seraient les antibiotiques à privilégier pour le traitement de l'OMSC en Angola. Cependant, compte tenu de la possible émergence de résistances, l'utilisation d'antiseptiques locaux tels que l'acétate d'aluminium, l'acide acétique ou l'acide borique semble davantage faisable.

Translated from English version into French by Suzane Assenat, proofread by Rodolphe Blet, through

TRANSLATORS WITHOUT BORDERS

Аэробные бактерии, связанные с хроническим гнойным средним отитом в Анголе

Фабиан Удден, Матуба Филип, Оке Реймер, Мария Пол, Эрика Матушек, Джон Тегерсторм, Свен Хаммершмидт, Тула Пелконен, Кристиан Риесбек

Аннотация

Справочная информация: Хронический гнойный средний отит (ХГСО) представляет собой серьёзную причину потери слуха у детей и является значительной проблемой здравоохранения на международном уровне, при этом присутствует тесная связь с условиями жизни, сопровождающимися дефицитом ресурсов. Местные антибиотики в сочетании с туалетом слухового прохода являются первостепенным методом лечения ХГСО, однако устойчивость к противомикробным препаратам, а также ограниченность доступа к антибиотикам создают трудности в некоторых районах. Цель настоящего исследования заключалась в выявлении возбудителей аэробных инфекций, ассоциированных с ХГСО в Анголе, тогда как общей целью было предоставление справочной информации для рекомендаций по местному лечению.

Методы: У 152 пациентов, страдающих ушными выделениями и разрывом барабанной перепонки, были собраны и культивированы образцы выделений из уха и носоглотки. По методу время-пролетной масс-спектрометрии с матрично-активированной лазерной десорбцией/ионизацией были выявлены разновидности бактерий, тогда как серотипы пневмококков были определены с использованием множественных полимеразных цепных реакций. Тестирование чувствительности к антимикробным препаратам проводилось в соответствии с требованиями Европейского комитета по определению чувствительности к антимикробным препаратам (EUCAST).

Результаты: Были собраны сто восемьдесят четыре образца выделений из уха и 151 мазков из носоглотки, что дало соответственно 534 и 289 индивидуальных изолятов. По всем пациентам уровень соответствия изолятов из двух ушей у больных двухсторонней формой заболевания составил 27,3% ; а также 9,3% при сравнении изолятов из мазков, полученных из носоглотки и выделений из уха соответственно. *Протей (Proteus* spp). (14,7%), *синегнойная палочка (Pseudomonas aeruginosa)* (13,2%) и *энтерококки (Enterococcus* spp). (8,8%) являлись

доминирующими патогенными организмами, изолированными из выделений из ушного прохода. Значительная часть остальных разновидностей микроорганизмов приходилась на энтеробактерии (Enterobacteriaceae) (23,5%). Приблизительно в 10% образцов выделений из носоглотки были выявлены пневмококки и *золотистый стафилококк (Staphylococcus aureus)* . Уровень устойчивости к хинолонам превысил 10% в случае энтеробактерий и находился на уровне 30, 8% у *золотистого стафилококка*; тогда как в 6,3% *синегнойной палочки* была обнаружена резистентность.
Выводы: Инфицированность среднего уха в случаях ХГСО является крайне полимикробной, а изоляты, обнаруженные в носоглотке, слабо соответствуют тем, которые были получены из выделений ушного прохода. В патогенных микроорганизмах, ассоциируемых с ХГСО в Анголе, преобладают грамоотрицательные бактерии, в том числе энтеробактерии и *синегнойная палочка,* однако также встречаются и грамположительные энтерококки. Основываясь на результатах тестирования чувствительности к антимикробным препаратам, местное введение хинолонов является предпочтительной антибактериальной терапией ХГСО в Анголе. При этом использование местных антисептиков, таких как ацетат алюминия, уксусная кислота или борная кислота, может представлять собой более выполнимый вариант вследствие вероятности возникновения устойчивости к антимикробным препаратам.

Translated from English version into Russian by Liudmila Tomanek, proofread by Ekaterina_Rugg, through

TRANSLATORS
WITHOUT BORDERS

Bacterias aeróbicas asociadas con otitis media supurativa crónica en Angola

Fabian Uddén, Matuba Filipe, Åke Reimer, Maria Paul, Erika Matuschek, John Thegerström, Sven Hammerschmidt, Tuula Pelkonen, Kristian Riesbeck.

Resumen

Antecedentes: La otitis media supurativa crónica (OMSC) es una importante causa de sordera parcial en niños y constituye un problema de salud grave en todo el mundo. Presenta una estrecha vinculación con las condiciones de vida marcadas por la escasez de recursos. Los antibióticos tópicos combinados con el lavado ótico son el tratamiento directo para la OMSC. Sin embargo, la resistencia bacteriana a los antibióticos y la disponibilidad limitada de estos representan un obstáculo en ciertas zonas. El objetivo de este estudio era definir los microorganismos patógenos aerobios asociados con la OMSC en Angola con el propósito general de proporcionar unos antecedentes que permitan elaborar recomendaciones para los tratamientos locales.
Metodología: Se recogieron y cultivaron muestras de otorrea y exudado nasofaríngeo de 152 pacientes con otorrea y perforación de la membrana timpánica. La identificación de las especies de bacterias se llevó a cabo mediante una espectrometría de masas MALTI-TOF y los neumococos se serotiparon utilizando reacciones en cadena de la polimerasa múltiple. El antibiograma se realizó siguiendo las indicaciones del EUCAST.
Resultados: Se recogieron 184 muestras de otorrea y 151 de exudado nasofaríngeo en las que se hallaron 534 y 289 cepas, respectivamente. En todos los pacientes, la tasa de correspondencia de las cepas de ambos oídos en pacientes con OMSC bilateral fue de un 27,3 % y de un 9,3 % al compararlas con las cepas del exudado nasofaríngeo y la otorrea, respectivamente. *Proteus* spp. (14,7 %), *Pseudomonas aeruginosa* (13,2 %) y *Enterococcus* spp. (8,8 %) eran los

microorganismos patógenos predominantes entre los que se aislaron a partir de la otorrea. Una gran parte del resto de especies pertenecían a la familiaEnterobacteriaceae(23,5 %). Se detectaron neumococos y *Staphylococcus aureus* en aproximadamente un 10 % de las muestras nasofaríngeas. La tasa de resistencia a los antibióticos quinolónicos superaba el 10 % entre las *Enterobacteriaceae* y era del 30,8 % en el caso del *Staphylococcus aureus*, mientras que, entre las *P. aeruginosa*, un 6,3 % mostraban resistencia.

Conclusiones: La infección del oído medio en la OMSC es altamente polimicrobiana y las cepas halladas en las muestras nasofaríngeas no se corresponden bien con las presentes en la otorrea. Entre los microorganismos patógenos asociados con la OMSC en Angola predominan los gramnegativos, incluyendo Enterobacteriaceae y *P. aeruginosa,* aunque que los enterococos grampositivos también son comunes. Teniendo en cuenta los resultados del antibiograma, las quinolonas tópicas serían los antibióticos preferidos para tratar la OMSC en Angola. No obstante, los antisépticos tópicos, como el acetato de aluminio, el ácido acético o el ácido bórico, podrían ser opciones más viables debido a la posible aparición de resistencias a los antibióticos.

Translated from English version into Spanish by Noelia Bernárdez, proofread by Iñaki Vega Bayo, through

TRANSLATORS
WITHOUT BORDERS
