# Supplementary material for: Aerobic bacteria associated with chronic suppurative otitis media in Angola
Source: Infect Dis Poverty. 2018 May 3;7:42. doi: 10.1186/s40249-018-0422-7 (PMC5932871; doi:10.1186/s40249-018-0422-7)
Supplement: Supplementary file 2 — Primer list and PCR cycling conditions. (PDF 126 kb) [file 40249_2018_422_MOESM2_ESM.pdf]

Additional file 2. Primer list and PCR cycling conditions.

List of primers used in mPCR for serotyping of pneumococci:

mPCR number	Target serotype(s)	Forward primer sequence	Reverse primer sequence	Product length (bp)
1	23B	CCACAATTAGCGCTATATTCATTCAATCG	GTCCACGCTGAATAAAATGAAGCTCCG	149
1	15B, 15C	ACCAGTTACGGAGTAAGATATGCAG	CGTGTCACATTACTACCTGTTCTTG	350
1	11A, 11D	GGACATGTTCAGGTGATTTCCCAATATAGTG	GATTATGAGTGTAATTTATTCCAACTTCTCCC	463
1	35F, 47F	GAACATAGTCGCTATTGTATTTTATTTAAAGCAA	GACTAGGAGCATTATTCCTAGAGCGAGTAAACC	517
1	35B	GATAAGTCTGTTGTGGAGACTTAAAAAGAATG	CTTTCCAGATAATTACAGGTATTCCTGAAGCAAG	677
2	21	CTATGGTTATTTCAACTCAATCGTCACC	GGCAAACTCAGACATAGTATAGCATAG	192
2	6A, 6B, 6C, 6D	AATTTGTATTTTATTCATGCCTATATCTGG	TTAGCGGAGATAATTTAAAATGATGACTA	250
2	3	ATGGTGTGATTTCTCCTAGATTGGAAAGTAG	CTTCTCCAATTGCTTACCAAGTGCAATAACG	371
2	19A	GAGAGATTCATAATCTTGCACTTAGCCA	CATAATAGCTACAAATGACTCATCGCC	566
2	23A	TATTCTAGCAAGTGACGAAGATGCG	CCAACATGCTTAAAAACGCTGCTTTAC	722
3	14	GAAATGTTACTTGGCGCAGGTGTCAGAATT	GCCAATACTTCTTAGTCTCTCAGATGAAT	189
3	19F	GTTAAGATTGCTGATCGATTAATTGATATCC	GTAATATGTCTTTAGGGCGTTTATGGCGATAG	304
3	23F	GTAACAGTTGCTGTAGAGGGAATTGGCTTTTC	CACAACACCTAACACTCGATGGCTATATGATTC	384
3	18A, 18B, 18C, 18F	CTTAATAGCTCTCATTATTCTTTTTTTAAGCC	TTATCTGTAAACCATATCAGCATCTGAAAC	573
3	17F	TTCGTGATGATAATTCCAATGATCAAACAAGAG	GATGTAACAAATTTGTAGCGACTAAGGTCTGC	693
3	9A, 9V	GGGTTCAAAGTCAGACAGTGAATCTTAA	CCATGAATGAAATCAACATTGTCAGTAGC	816
4	7C, 7B, 40	CTATCTCAGTCATCTATTGTTAAAGTTTACGACGGGA	GAACATAGATGTTGAGACATCTTTTGTAATTTC	260
4	33F, 33A, 37	GAAGGCAATCAATGTGATTGTGTCGCG	CTTCAAAATGAAGATTATAGTACCCTTCTAC	338
4	15A, 15F	ATTAGTACAGCTGCTGGAATATCTCTTC	GATCTAGTGAACGTACTATTCCAAAC	434
4	9N, 9L	GAACTGAATAAGTCAGATTTAATCAGC	ACCAAGATCTGACGGGCTAATCAAT	516
4	10A	GGTGTAGATTTACCATTAGTGTCGGCAGAC	GAATTTCTTCTTTAAGATTCGGATATTTCTC	628
4	16F	GAATTTTTCAGGCGTGGGTGTTAAAAG	CAGCATATAGCACCGCTAAGCAAATA	717
5	1	CTCTATAGAATGGAGTATATAAACTATGGTTA	CCAAAGAAAATACTAACATTATCACAATATTGGC	280
5	5	ATACCTACACAACTTCTGATTATGCCTTTGTG	GCTCGATAAACATAATCAATATTTGAAAAAGTATG	362
5	4	CTGTTACTTGTTCTGGACTCTCGATAATTGG	GCCCACTCCTGTTAAAATCCTACCCGCATTG	430
5	7F, 7A	TCCAAACTATTACAGTGGGAATTACGG	ATAGGAATTGAGATTGCCAAAGCGAC	599
5	31	GGAAGTTTTCAAGGATATGATAGTGGTGGTGC	CCGAATAATATATTCAATATATTCCTACTC	701
6	8	GAAGAAACGAAACTGTCAGAGCATTTACAT	CTATAGATACTAGTAGAGCTGTTCTAGTCT	201
6	2	TATCCCAGTTCAATATTTCTCCACTACACC	ACACAAAATATAGGCAGAGAGAGACTACT	290
6	12A, 12B, 12C, 12F, 44, 46	GCAACAAACGGCGTGAAAGTAGTTG	CAAGATGAATATCACTACCAATAACAAAAC	376
6	20	GAGCAAGAGTTTTTCACCTGACAGCGAGAAG	CTAAATTCCTGTAATTTAGCTAAAACTCTTATC	514
6	22	GAGTATAGCCAGATTATGGCAGTTTTATTGTC	CTCCAGCACTTGCGCTGGAAACAACAGACAAC	643
All reactions	*cpsA* (positive control)	GCAGTACAGCAGTTTGTTGGACTGACC	GAATATTTTCATTATCAGTCCCAGTC	160

PCR cycling conditions:

	Predenaturation:	95°C for 15 min
	Denaturation:	94°C for 30 s
35 X	Annealing:	54°C for 90 s
	Extension:	72°C for 60 s
	Final extension:	72°C for 10 min
