# Supplementary material for: Aerobic bacteria associated with chronic suppurative otitis media in Angola
Source: Infect Dis Poverty. 2018 May 3;7:42. doi: 10.1186/s40249-018-0422-7 (PMC5932871; doi:10.1186/s40249-018-0422-7)
Supplement: Supplementary file 3 — Full patient list. (PDF 258 kb) [file 40249_2018_422_MOESM3_ESM.pdf]

Additional file 3. Full patient list.

List of all patients including data on gender, age and province of residence as well as microbiological findings in all samples.

Patient count	Gender	Age	Province	Isolates in ear discharge sample 1	Isolates in ear discharge sample 2 (bilateral CSOM)	Isolates in nasopharyngeal sample
1	Female	1 mo	Luanda	*Enterococcus faecalis, Proteus mirabilis, Pseudomonas aeruginosa*	-	*Staphylococcus aureus*
2	Male	2 mo	Luanda	*Enterococcus faecalis, Arthrobacter cumminsii, Proteus mirabilis, Alcaligenes faecalis*	-	*Moraxella catarrhalis, Haemophilus influenza, Streptococcus pneumoniae*
3	Male	6 mo	-	*Streptococcus pyogenes, Staphylococcus aureus*	-	*Streptococcus pneumoniae, Streptococcus pyogenes, Moraxella nonliquefaciens, Staphylococcus epidermidis*
4	Female	7 mo	Luanda	*Enterococcus avium, Morganella morganii, Proteus mirabilis, Alcaligenes faecalis, Providencia stuartii*	-	*Proteus mirabilis, Staphylococcus saprophyticus*
5	Female	8 mo	Luanda	*Escherichia coli*	-	*Streptococcus pneumoniae, Haemophilus influenzae*
6	Female	8 mo	Lunda Sul	*Enterococcus hirae, Weissella confusa, Pseudomonas stutzeri, Corynebacterium amycolatum*	*Streptococcus anginosus, Streptococcus pyogenes, Staphylococcus epidermidis, Corynebacterium amycolatum*	*Enterococcus faecium, Macrococcus caseolyticus*
7	Male	9 mo	Luanda	*Staphylococcus epidermidis, Streptococcus lutetiensis*	No growth	*Haemophilus influenzae, Moraxella catarrhalis, Streptococcus pneumoniae*
8	Male	1 yr	Luanda	*Staphylococcus haemolyticus, Pseudomonas aeruginosa, Providencia stuartii*	-	*Acinetobacter radioresistens*
9	Female	1 yr 1 mo	Luanda	*Enterococcus faecalis, Klebsiella pneumoniae, Citrobacter freundii, Providencia stuartii, Proteus mirabilis*	-	*Staphylococcus aureus, Pseudomonas stutzeri*

10	Female	1 yr 2 mo	Luanda	*Morganella morganii, Pseudomonas aeruginosa, Klebsiella oxytoca*	-	*Moraxella catarrhalis, Streptococcus pneumoniae*
11	Male	1 yr 7 mo	Luanda	*Klebsiella oxytoca, Alcaligenes faecalis, Pseudomonas aeruginosa*	-	*Staphylococcus aureus, Streptococcus pneumoniae, Moraxella catarrhalis*
12	Male	1 yr 8 mo	Luanda	*Staphylococcus aureus, Enterococcus avium, Proteus mirabilis*	-	*Proteus mirabilis, Staphylococcus aureus, Staphylococcus saprophyticus*
13	Female	1 yr 8 mo	Luanda	*Streptococcus constellatus, Enterococcus faecalis, Providencia rettgeri, Proteus mirabilis, Kocuria kristinae*	*Streptococcus constellatus, Lactococcus lactis, Proteus mirabilis, Klebsiella oxytoca*	*Streptococcus pneumoniae, Proteus mirabilis*
14	Female	1 yr 8 mo	Luanda	*Clavispora lusitaniae*	*Streptococcus pyogenes, Proteus mirabilis, Candida albicans*	*Streptococcus pneumoniae, Staphylococcus saprophyticus, Pseudomonas stutzeri*
15	Male	2 yr	Luanda	*Proteus mirabilis, Pseudomonas aeruginosa, Achromobacter xylosoxidans*	-	*Pseudomonas putida*
16	Male	2 yr 2 mo	Namibe	*Weeksella virosa, Proteus mirabilis*	-	*Enterococcus faecium, Pseudomonas stutzeri*
17	Male	2 yr 2 mo	Lunda Sul	*Enterococcus faecalis, Enterococcus avium, Proteus mirabilis*	-	*Enterococcus avium*
18	Male	2 yr 4 mo	Luanda	*Providencia stuartii, Corynebacterium amycolatum, Streptococcus pneumoniae, Morganella morganii*	*Staphylococcus aureus, Corynebacterium amycolatum, Streptococcus pyogenes*	*Moraxella catarrhalis*
19	Female	2 yr 6 mo	Luanda	*Proteus mirabilis, Pseudomonas aeruginosa*	-	*Streptococcus pneumoniae, Pseudomonas* sp.
20	Female	2 yr 10 mo	Lunda Sul	*Staphylococcus xylosus, Candida parapsilosis*	-	*Enterococcus hirae*
21	Male	2 yr 10 mo	Luanda	*Proteus mirabilis, Pseudomonas aeruginosa*	-	*Staphylococcus saprophyticus*

22	Male	3 yr 2 mo	Luanda	*Enterococcus raffinosus, Kerstersia gyiorum, Morganella morganii, Pseudomonas otitidis*	-	*Streptococcus mitis*
23	Male	3 yr 2 mo	Lunda Sul	No growth	-	*Streptococcus pyogenes, Corynebacterium amycolatum, Staphylococcus aureus*
24	Female	3 yr 6 mo	Luanda	*Streptococcus constellatus, Providencia stuartii, Kerstersia gyiorum*	-	*Streptococcus pneumoniae, Moraxella catarrhalis*
25	Male	3 yr 7 mo	Luanda	*Corynebacterium striatum, Citrobacter amalonaticus, Proteus mirabilis, Alcaligenes faecalis*	*Corynebacterium striatum, Proteus mirabilis, Enterococcus avium, Alcaligenes faecalis*	*Streptococcus pneumoniae, Alcaligenes faecalis, Staphylococcus saprophyticus*
26	Male	3 yr 7 mo	Luanda	*Staphylococcus aureus, Pseudomonas aeruginosa, Morganella morganii*	*Corynebacterium amycolatum, Pseudomonas aeruginosa, Staphylococcus aureus, Streptococcus intermedius*	*Streptococcus pneumoniae*
27	Female	3 yr 7 mo	Luanda	*Morganella morganii, Citrobacter freundii*	*Enterococcus faecalis, Enterobacter cloacae, Escherichia coli*	*Staphylococcus aureus, Pseudomonas stutzeri, Staphylococcus hominis*
28	Male	3 yr 10 mo	Lunda Sul	*Enterococcus faecalis, Proteus mirabilis*	-	*Pantoea agglomerans*
29	Male	4 yr 2 mo	Luanda	*Staphylococcus equorum*	*Citrobacter koseri, Providencia stuartii*	*Staphylococcus sciuri*
30	Female	4 yr 5 mo	Luanda	*Staphylococcus aureus*	*Staphylococcus aureus*	*Haemophilus influenzae, Moraxella catarrhalis, Streptococcus pneumoniae*
31	Male	4 yr 7 mo	Luanda	*Enterococcus faecalis, Proteus mirabilis, Citrobacter freundii, Pseudomonas aeruginosa, Providencia rettgeri*	-	*Staphylococcus xylosus*
32	Female	4 yr 10 mo	Luanda	*Arthrobacter cumminsii, Staphylococcus epidermidis, Pseudomonas aeruginosa, Providencia stuartii*	-	*Staphylococcus xylosus, Moraxella catarrhalis, Raoultella ornithinolytica, Acinetobacter radioresistens, Streptococcus pneumoniae, Pseudomonas stutzeri*
33	Male	4 yr 10 mo	Luanda	*Enterobacter cloacae, Pseudomonas aeruginosa*	-	*Staphylococcus epidermidis*

34	Male	5 yr	Lunda Sul	*Aerococcus viridans, Streptococcus pneumoniae, Arthrobacter polychromogenes*	-	*Morganella morganii, Alcaligenes faecalis*
35	Male	5 yr 1 mo	-	No growth	-	*Streptococcus pneumoniae*
36	Male	5 yr 2 mo	Luanda	*Enterococcus avium, Providencia stuartii, Kerstersia gyiorum*	-	*Streptococcus pneumoniae*
37	Female	5 yr 5 mo	Luanda	*Staphylococcus saprophyticus, Pseudomonas aeruginosa, Alcaligenes faecalis*	-	*Streptococcus pneumoniae, Staphylococcus saprophyticus, Moraxella catarrhalis*
38	Male	5 yr 6 mo	Lunda Sul	*Escherichia coli, Enterobacter asburiae, Raoultella ornithinolytica, Enterobacter cloacae, Serratia marcescens, Acinetobacter nosocomialis*	-	*Arthrobacter polychromogenes*
39	Female	5 yr 6 mo	Luanda	*Enterococcus faecalis*	-	*Enterococcus faecalis, Pseudomonas stutzeri*
40	Male	5 yr 7 mo	Lunda Sul	*Proteus vulgaris, Enterococcus avium*	*Enterococcus raffinosus, Proteus vulgaris, Pseudomonas aeruginosa*	*Arthrobacter polychromogenes, Alcaligenes faecalis*
41	Female	5 yr 8 mo	Luanda	*Corynebacterium striatum, Kerstersia gyiorum, Pseudomonas aeruginosa, Pseudomonas otitidis*	-	*Staphylococcus epidermidis*
42	Male	5 yr 9 mo	Luanda	*Escherichia coli, Pseudomonas aeruginosa, Corynebacterium amycolatum*	-	*Staphylococcus aureus*
43	Female	6 yr	Luanda	*Enterococcus faecalis, Providencia stuartii, Pseudomonas aeruginosa, Proteus mirabilis*	*Enterococcus faecalis, Providencia stuartii, Pseudomonas aeruginosa, Proteus mirabilis*	*Streptococcus pneumoniae*
44	Female	6 yr	Luanda	*Staphylococcus sciuri, Pseudomonas aeruginosa, Pseudomonas aeruginosa*	-	*Acinetobacter lwoffii, Staphylococcus saprophyticus*
45	Male	6 yr 5 mo	Luanda	*Proteus mirabilis*	-	*Leuconostoc lactis*

46	Male	6 yr 8 mo	Zaire	*Morganella morganii, Proteus mirabilis, Providencia rettgeri, Enterococcus raffinosus*	-	*Staphylococcus saprophyticus, Pseudomonas stutzeri*
47	Male	6 yr 10 mo	Lunda Sul	*Arthrobacter polychromogenes, Staphylococcus haemolyticus, Pseudomonas fulva, Klebsiella pneumoniae*	-	*Enterococcus faecium, Pantoea agglomerans*
48	Male	6 yr 10 mo	Zaire	*Staphylococcus haemolyticus, Pseudomonas aeruginosa, Achromobacter* sp.	-	*Staphylococcus saprophyticus, Staphylococcus xylosus*
49	Male	7 yr 1 mo	Luanda	*Streptococcus dysgalactiae, Pseudomonas aeruginosa, Pseudomonas fulva*	-	*Enterococcus faecium, Serratia marcescens, Pseudomonas synxantha*
50	Female	7 yr 2 mo	Lunda Sul	*Enterococcus hirae, Pseudomonas extremorientalis, Citrobacter freundii*	-	*Candida parapsilosis*
51	Male	8 yr	Luanda	*Enterococcus faecalis, Alcaligenes faecalis, Citrobacter freundii, Proteus mirabilis*	-	*Enterobacter asburiae, Candida parapsilosis*
52	Female	8 yr 4 mo	Luanda	*Pseudomonas mendocina, Pseudomonas aeruginosa, Achromobacter xylosoxidans*	-	*Streptococcus pneumoniae, Citrobacter koseri*
53	Female	8 yr 5 mo	Lunda Sul	*Enterobacter cloacae, Staphylococcus aureus, Morganella morganii, Enterobacter asburiae*	*Morganella morganii, Staphylococcus aureus*	*Leuconostoc citreum*
54	Male	9 yr 2 mo	Luanda	*Candida tropicalis, Enterococcus faecalis, Klebsiella oxytoca, Pseudomonas aeruginosa, Stenotrophomonas maltophilia*	-	No growth
55	Male	9 yr 6 mo	Luanda	*Corynebacterium striatum, Proteus mirabilis*	-	*Staphylococcus aureus, Streptococcus pneumoniae*
56	Male	9 yr 6 mo	Luanda	*Enterococcus faecalis, Enterococcus avium, Pseudomonas aeruginosa, Alcaligenes faecalis, Escherichia coli, Proteus vulgaris/penneris*	-	*Staphylococcus saprophyticus, Staphylococcus sciuri*
57	Male	9 yr 8 mo	Lunda Sul	*Arthrobacter polychromogenes*	-	*Arthrobacter polychromogenes*

58	Male	10 yr	Luanda	*Enterococcus faecium, Proteus mirabilis*	-	*Staphylococcus saprophyticus*
59	Female	10 yr 1 mo	Luanda	*Staphylococcus saprophyticus, Staphylococcus haemolyticus, Pseudomonas aeruginosa, Staphylococcus aureus*	-	*Corynebacterium propinquum*
60	Male	10 yr 1 mo	Luanda	*Corynebacterium diphtheriae, Pseudomonas aeruginosa*	*Streptococcus constellatus, Corynebacterium diphtheriae, Pseudomonas aeruginosa, Providencia stuartii*	*Staphylococcus saprophyticus*
61	Female	10 yr 2 mo	Namibe	*Proteus mirabilis, Kerstersia gyiorum, Providencia rettgeri*	-	*Streptococcus pneumoniae, Pseudomonas stutzeri*
62	Male	10 yr 11 mo	Luanda	*Corynebacterium amycolatum, Aeromonas caviae*	-	*Staphylococcus saprophyticus, Staphylococcus aureus*
63	Male	10 yr 11 mo	Luanda	*Enterococcus hirae, Enterobacter cloacae*	-	*Pseudomonas plecoglossicida, Wickerhamomyces anomalus*
64	Female	11 yr 3 mo	Luanda	*Corynebacterium amycolatum, Arthrobacter cumminsii, Proteus mirabilis, Providencia stuartii*	*Weeksella virosa, Arthrobacter cumminsii, Bordetella trematum, Aerococcus viridans*	*Staphylococcus saprophyticus, Staphylococcus xylosus, Pseudomonas stutzeri*
65	Female	11 yr 4 mo	Luanda	*Arthrobacter cumminsii, Proteus mirabilis, Klebsiella pneumoniae, Pseudomonas aeruginosa*	-	*Staphylococcus aureus*
66	Male	11 yr 10 mo	Luanda	*Morganella morganii, Klebsiella oxytoca, Providencia rettgeri, Proteus mirabilis*	-	*Staphylococcus xylosus, Staphylococcus saprophyticus, Enterococcus faecalis, Staphylococcus aureus, Enterobacter cloacae*
67	Male	12 yr 3 mo	Luanda	*Arthrobacter cumminsii, Staphylococcus xylosus, Proteus mirabilis, Escherichia coli*	-	*Staphylococcus xylosus, Pseudomonas fulva, Staphylococcus sciuri*
68	Female	12 yr 4 mo	Luanda	*Morganella morganii, Corynebacterium striatum, Proteus mirabilis, Citrobacter koseri*	*Morganella morganii, Corynebacterium striatum, Achromobacter xylosoxidans, Citrobacter koseri*	*Staphylococcus epidermidis*
69	Male	12 yr 11 mo	Lunda Sul	*Enterococcus avium, Proteus mirabilis*	*Enterococcus avium, Proteus mirabilis*	*Alcaligenes faecalis*

70	Male	13 yr	Luanda	*Staphylococcus epidermidis, Kerstersia gyiorum, Providencia stuartii*	*Morganella morganii, Kerstersia gyiorum, Proteus mirabilis*	*Pseudomonas plecoglossicida, Staphylococcus epidermidis*
71	Male	13 yr 6 mo	Luanda	*Staphylococcus saprophyticus, Staphylococcus xylosus*	-	*Staphylococcus epidermidis, Staphylococcus saprophyticus, Pseudomonas stutzeri*
72	Male	13 yr 8 mo	Zaire	*Morganella morganii, Enterobacter cloacae, Citrobacter freundii, Pseudomonas aeruginosa*	-	*Staphylococcus aureus*
73	Male	13 yr 8 mo	Lunda Sul	*Enterococcus avium, Proteus mirabilis, Providencia rettgeri*	*Enterococcus avium, Proteus mirabilis, Providencia rettgeri*	*Proteus mirabilis*
74	Female	13 yr 10 mo	Luanda	*Staphylococcus haemolyticus, Staphylococcus saprophyticus, Gemella morbillorum, Proteus mirabilis, Pseudomonas aeruginosa, Alcaligenes faecalis, Citrobacter koseri*	-	*Staphylococcus saprophyticus, Staphylococcus epidermidis, Acinetobacter radioresistens*
75	Female	13 yr 10 mo	Luanda	*Gemella morbillorum, Corynebacterium aurimucosum, Proteus mirabilis, Arcanobacterium haemolyticum*	*Arcanobacterium haemolyticum, Corynebacterium aurimucosum, Proteus mirabilis*	*Streptococcus pneumoniae, Pseudomonas fluorescens*
76	Male	13 yr 11 mo	Lunda Sul	*Alcaligenes faecalis, Streptococcus constellatus, Pseudomonas aeruginosa*	-	*Arthrobacter histidinolovorans, Arthrobacter polychromogenes*
77	Male	14 yr 1 mo	Lunda Sul	*Morganella morganii, Proteus mirabilis*	-	*Proteus mirabilis, Staphylococcus saprophyticus, Enterococcus faecium*
78	Male	14 yr 3 mo	Luanda	*Corynebacterium striatum, Proteus mirabilis*	-	*Streptococcus pneumoniae, Staphylococcus saprophyticus, Staphylococcus epidermidis, Pseudomonas stutzeri*
79	Male	14 yr 7 mo	Luanda	*Enterococcus faecalis, Arthrobacter cumminsii, Citrobacter koseri, Neisseria meningitidis*	-	*Staphylococcus aureus*
80	Female	14 yr 7 mo	Luanda	*Pseudomonas aeruginosa*	-	*Shewanella putrefaciens, Streptococcus pneumoniae*
81	Male	14 yr 10 mo	Lunda Sul	*Micrococcus terreus, Candida haemulonii*	-	No growth

82	Male	15 yr 2 mo	Luanda	*Proteus mirabilis*	-	*Streptococcus pneumoniae, Staphylococcus saprophyticus, Pseudomonas stutzeri*
83	Female	15 yr 7 mo	Luanda	*Arthrobacter cumminsii, Providencia stuartii, Proteus mirabilis*	-	*Corynebacterium propinquum*
84	Male	15 yr 11 mo	Luanda	*Proteus mirabilis*	-	*Streptococcus pyogenes, Acinetobacter radioresistens, Pseudomonas stutzeri*
85	Male	16 yr 1 mo	Luanda	*Proteus mirabilis, Providencia rettgeri, Kerstersia gyiorum*	*Providencia rettgeri, Proteus mirabilis*	*Staphylococcus epidermidis, Pseudomonas plecoglossicida*
86	Male	16 yr 6 mo	Luanda	*Providencia stuartii, Pseudomonas aeruginosa*	-	*Staphylococcus aureus*
87	Male	16 yr 11 mo	Luanda	*Streptococcus pyogenes, Staphylococcus aureus*	-	*Streptococcus pneumoniae, Pseudomonas stutzeri*
88	Male	17 yr 11 mo	Luanda	*Corynebacterium aurimucosum, Providencia stuartii, Proteus mirabilis, Kerstersia gyiorum*	*Staphylococcus xylosus, Pseudomonas aeruginosa, Aeromonas caviae*	*Staphylococcus xylosus, Staphylococcus sciuri, Staphylococcus saprophyticus, Pseudomonas fulva*
89	Male	18 yr 2 mo	Luanda	*Pseudomonas aeruginosa, Achromobacter xylosoxidans*	-	-
90	Male	18 yr 8 mo	Luanda	*Corynebacterium amycolatum, Proteus mirabilis*	-	*Staphylococcus epidermidis, Staphylococcus saprophyticus, Proteus mirabilis*
91	Male	18 yr 10 mo	Lunda Sul	*Enterococcus avium, Morganella morganii, Proteus mirabilis, Providencia rettgeri*	-	*Lactococcus lactis, Staphylococcus saprophyticus*
92	Female	19 yr	Zaire	*Staphylococcus sciuri, Pseudomonas aeruginosa*	-	*Staphylococcus aureus*
93	Female	19 yr	Luanda	*Pseudomonas aeruginosa, Pseudomonas aeruginosa*	*Staphylococcus haemolyticus, Pseudomonas aeruginosa, Pseudomonas aeruginosa*	*Staphylococcus xylosus, Pseudomonas stutzeri*

94	Male	19 yr 1 mo	-	*Staphylococcus aureus*	-	*Pseudomonas stutzeri*
95	Female	19 yr 8 mo	Luanda	*Enterococcus faecalis, Proteus mirabilis, Pseudomonas aeruginosa*	-	*Staphylococcus aureus, Pseudomonas stutzeri*
96	Male	19 yr 10 mo	Lunda Sul	*Providencia rettgeri, Proteus mirabilis*	-	*Staphylococcus cohnii, Staphylococcus epidermidis*
97	Female	20 yr 1 mo	Luanda	*Enterococcus avium, Proteus mirabilis, Kerstersia gyiorum*	-	*Staphylococcus saprophyticus, Pseudomonas fulva*
98	Male	20 yr 4 mo	Luanda	*Pseudomonas aeruginosa, Pseudomonas aeruginosa, Pseudomonas aeruginosa*	-	*Proteus vulgaris*
99	Male	20 yr 4 mo	Luanda	*Morganella morganii, Pseudomonas aeruginosa, Citrobacter freundii, Providencia rettgeri, Proteus mirabilis*	*Enterococcus faecalis, Morganella morganii, Proteus mirabilis, Providencia rettgeri, Pseudomonas aeruginosa, Alcaligenes faecalis*	*Staphylococcus epidermidis, Pseudomonas plecoglossicida*
100	Male	20 yr 5 mo	Luanda	*Corynebacterium amycolatum, Providencia stuartii, Pseudomonas aeruginosa, Citrobacter koseri, Proteus mirabilis*	-	*Staphylococcus saprophyticus, Aerococcus viridans, Proteus mirabilis*
101	Male	21 yr 3 mo	Luanda	*Staphylococcus aureus, Staphylococcus saprophyticus, Enterobacter cloacae, Stenotrophomonas maltophilia*	-	*Pseudomonas plecoglossicida, Pseudomonas putida*
102	Female	21 yr 8 mo	Luanda	*Enterococcus faecium, Kerstersia gyiorum, Proteus mirabilis*	*Enterococcus faecalis, Proteus mirabilis, Escherichia coli, Bordetella trematum*	*Staphylococcus saprophyticus, Staphylococcus epidermidis*
103	Male	22 yr 4 mo	Luanda	*Morganella morganii, Alcaligenes faecalis, Proteus mirabilis, Pseudomonas aeruginosa, Klebsiella pneumoniae*	-	*Pseudomonas fluorescens, Staphylococcus epidermidis*
104	Female	22 yr 5 mo	Luanda	*Streptococcus oralis, Streptococcus anginosus, Corynebacterium striatum*	-	*Staphylococcus saprophyticus, Acinetobacter* sp.
105	Male	23 yr 3 mo	Luanda	*Staphylococcus aureus, Staphylococcus saprophyticus, Pseudomonas aeruginosa*	-	*Pseudomonas fulva*

106	Female	23 yr 3 mo	Luanda	*Klebsiella pneumoniae, Alcaligenes faecalis, Citrobacter freundii*	-	*Staphylococcus epidermidis, Streptococcus pneumoniae, Alcaligenes faecalis*
107	Male	24 yr 7 mo	Luanda	*Staphylococcus haemolyticus, Corynebacterium amycolatum*	-	*Staphylococcus epidermidis*
108	Male	25 yr	Luanda	*Providencia rettgeri, Pseudomonas aeruginosa*	-	*Staphylococcus epidermidis*
109	Female	27 yr 10 mo	Luanda	*Enterococcus faecalis, Providencia stuartii, Achromobacter denitrificans, Pseudomonas putida, Stenotrophomonas maltophilia*	-	*Staphylococcus saprophyticus, Pseudomonas fulva*
110	Male	29 yr 4 mo	Luanda	*Micrococcus luteus, Dermabacter hominis*	-	*Staphylococcus epidermidis*
111	Female	30 yr 7 mo	Luanda	*Proteus mirabilis, Acinetobacter baumannii*	-	*Proteus mirabilis, Staphylococcus hominis*
112	Female	31 yr 5 mo	Luanda	*Pseudomonas aeruginosa, Morganella morganii*	*Pseudomonas aeruginosa, Morganella morganii*	*Staphylococcus saprophyticus*
113	Male	31 yr 8 mo	Lunda Sul	*Staphylococcus sciuri, Proteus mirabilis*	*Staphylococcus sciuri, Proteus mirabilis*	*Dietzia maris*
114	Male	33 yr 2 mo	Luanda	*Corynebacterium amycolatum, Proteus mirabilis*	*Staphylococcus epidermidis, Corynebacterium amycolatum*	*Streptococcus pneumoniae*
115	Female	35 yr 7 mo	Luanda	*Enterococcus casseliflavus, Escherichia coli, Pseudomonas mendocina*	-	*Staphylococcus hominis*
116	Female	36 yr 4 mo	Luanda	*Arthrobacter cumminsii, Proteus mirabilis*	-	No growth
117	Male	36 yr 4 mo	Luanda	*Klebsiella oxytoca, Candida tropicalis*	-	*Staphylococcus epidermidis, Staphylococcus hominis, Pseudomonas oryzihabitans*

118	Male	38 yr 7 mo	Luanda	*Providencia stuartii, Pseudomonas aeruginosa*	-	*Staphylococcus epidermidis, Lactobacillus salivarius*
119	Male	38 yr 9 mo	Luanda	*Pseudomonas aeruginosa, Pseudomonas aeruginosa, Kocuria* sp., *Staphylococcus cohnii*	-	*Staphylococcus epidermidis*
120	Male	39 yr 10 mo	Luanda	*Gemella morbillorum, Staphylococcus capitis, Escherichia hermannii*	-	*Staphylococcus caprae, Staphylococcus haemolyticus, Klebsiella pneumoniae*
121	Male	40 yr 6 mo	Luanda	*Klebsiella pneumoniae, Pseudomonas aeruginosa, Lodderomyces elongisporus*	-	*Pseudomonas fulva, Pseudomonas plecoglossicida, Pseudomonas fluorescens, Pseudomonas stutzeri*
122	Male	40 yr 10 mo	Luanda	*Proteus mirabilis, Citrobacter freundii*	-	*Staphylococcus xylosus*
123	Male	42 yr 8 mo	Luanda	*Staphylococcus saprophyticus, Staphylococcus epidermidis, Proteus mirabilis, Alcaligenes faecalis, Globicatella sulfidifaciens*	-	*Pseudomonas putida, Staphylococcus saprophyticus*
124	Female	43 yr 11 mo	Luanda	*Globicatella sulfidifaciens, Pseudomonas aeruginosa, Klebsiella pneumoniae, Proteus mirabilis, Pseudomonas aeruginosa, Bordetella trematum*	-	*Proteus mirabilis*
125	Female	44 yr 4 mo	Zaire	*Staphylococcus haemolyticus, Pseudomonas monteilii*	-	*Enterobacter aerogenes, Staphylococcus aureus, Staphylococcus xylosus, Pseudomonas fulva*
126	Female	44 yr 5 mo	Lunda Sul	*Proteus mirabilis*	-	*Pantoea agglomerans, Proteus mirabilis, Pantoea agglomerans*
127	Male	46 yr 3 mo	Luanda	*Enterococcus faecalis, Providencia stuartii, Morganella morganii, Kerstersia gyiorum*	-	*Pseudomonas stutzeri, Staphylococcus epidermidis*
128	Male	46 yr 7 mo	Luanda	*Arthrobacter cumminsii, Alcaligenes faecalis, Providencia stuartii*	-	No growth
129	Female	47 yr 3 mo	Luanda	*Staphylococcus epidermidis, Staphylococcus saprophyticus, Klebsiella pneumoniae, Acinetobacter baumannii, Kodamaea ohmeri*	-	*Staphylococcus xylosus*

130	Female	51 yr 8 mo	Luanda	*Candida metapsilosis*	-	*Staphylococcus saprophyticus, Candida metapsilosis*
131	Female	54 yr 11 mo	Luanda	*Staphylococcus cohnii,* fungus	-	*Staphylococcus xylosus*
132	Female	56 yr	Zaire	*Enterococcus faecalis, Proteus mirabilis, Klebsiella pneumoniae*	-	*Pseudomonas plecoglossicida, Staphylococcus sciuri*
133	Female	62 yr 1 mo	Luanda	*Staphylococcus haemolyticus, Escherichia coli*	*Enterobacter cloacae, Staphylococcus epidermidis*	*Staphylococcus epidermidis*
134	Female	62 yr 7 mo	Luanda	*Providencia stuartii, Corynebacterium freneyi, Staphylococcus haemolyticus*	*Providencia stuartii, Corynebacterium freneyi, Staphylococcus haemolyticus*	*Staphylococcus aureus, Staphylococcus arlettae*
135	Male	76 yr 1 mo	Zaire	*Enterobacter cloacae, Corynebacterium amycolatum, Escherichia hermannii*	-	*Staphylococcus aureus, Stenotrophomonas maltophilia, Staphylococcus cohnii, Staphylococcus epidermidis*
136	Male	77 yr 3 mo	Luanda	*Pseudomonas aeruginosa*	*Dermabacter hominis, Proteus mirabilis, Pseudomonas aeruginosa*	*Proteus mirabilis, Aerococcus viridans, Staphylococcus sciuri*
137	-	-	-	*Enterococcus faecium, Alcaligenes faecalis*	-	*Enterococcus hirae*
138	Male	-	Luanda	*Morganella morganii, Alcaligenes faecalis, Proteus mirabilis*	-	*Pseudomonas plecoglossicida, Staphylococcus sciuri*
139	Male	-	Luanda	*Candida parapsilosis*	-	*Pseudomonas stutzeri, Staphylococcus saprophyticus, Staphylococcus epidermidis*
140	-	-	-	*Morganella morganii, Providencia stuartii, Pseudomonas aeruginosa*	-	*Pseudomonas fulva, Klebsiella oxytoca, Staphylococcus aureus, Staphylococcus saprophyticus*
141	Male	-	Luanda	*Arthrobacter cumminsii, Pseudomonas aeruginosa, Providencia rettgeri, Proteus mirabilis*	-	*Staphylococcus epidermidis, Staphylococcus aureus*

142	Female	-	Luanda	*Corynebacterium aurimucosum, Escherichia coli*	-	*Streptococcus pneumoniae*
143	Male	-	Luanda	*Stenotrophomonas maltophilia, Pseudomonas aeruginosa, Providencia stuartii, Alcaligenes faecalis*	-	*Moraxella nonliquefaciens*
144	Female	-	Luanda	*Klebsiella pneumoniae, Pseudomonas aeruginosa*	-	*Staphylococcus aureus, Pseudomonas fulva*
145	-	-	-	*Providencia stuartii, Pseudomonas aeruginosa, Proteus mirabilis*	*Citrobacter koseri, Pseudomonas aeruginosa, Providencia stuartii, Alcaligenes faecalis*	*Streptococcus pneumoniae*
146	-	-	-	*Streptococcus pyogenes, Escherichia coli, Pseudomonas aeruginosa, Achromobacter denitrificans*	-	*Pseudomonas xanthomarina, Staphylococcus hominis*
147	Male	-	Lunda Sul	*Enterococcus faecalis, Proteus mirabilis*	-	*Macrococcus caseolyticus, Lactococcus lactis, Arthrobacter gandavensis*
148	Male	-	Lunda Sul	*Candida parapsilosis, Citrobacter freundii*	-	*Streptococcus pneumoniae, Arthrobacter polychromogenes*
149	-	-	-	*Streptococcus anginosus, Enterococcus faecalis, Pseudomonas aeruginosa, Pseudomonas aeruginosa*	-	*Moraxella catarrhalis, Corynebacterium propinquum*
150	Male	-	Lunda Sul	*Enterococcus raffinosus, Proteus mirabilis*	-	*Alcaligenes faecalis, Arthrobacter gandavensis*
151	Female	-	Luanda	*Arthrobacter cumminsii, Corynebacterium aurimucosum, Proteus mirabilis*	-	*Streptococcus pneumoniae, Staphylococcus aureus, Haemophilus influenzae*
152	Female	-	Luanda	*Proteus mirabilis, Proteus mirabilis, Pseudomonas aeruginosa*	-	*Staphylococcus epidermidis, Staphylococcus aureus, Staphylococcus haemolyticus*
